# Allelic frequencies of 22 short tandem repeats loci and tri-allelic patterns of Penta D and TPOX identified in Gabonese population

**DOI:** 10.1038/s41598-023-47395-z

**Published:** 2023-11-15

**Authors:** Elisabeth Lendoye, Landry Erick Mombo, Marie-Andree N’Negue ep. Mezui-Mbeng, Opheelia Makoyo Komba, Edgard Brice Ngoungou, Felix Ovono Abessolo, Joel Fleury Djoba Siawaya, Juliane Alt-Mörbe

**Affiliations:** 1 DNA-LAB Gabon Clinic, Libreville, Gabon; 2 Chemistry-Biochemistry Service, Department of Fundamental and Mixed Sciences, Faculty of Medicine, University of Health Sciences, Libreville, Gabon; 3 Laboratory Service, Mother, and Child University Hospital – Jeanne Ebori Foundation, Libreville, Gabon; 4 Laboratory of Molecular and Cellular Biology (LABMC), University of Sciences and Technology of Masuku (USTM), Franceville, Gabon; 5 Gynaecology-Obstetric and IVF Service, Mother, and Child Teaching Hospital – Jeanne Ebori Foundation, Libreville, Gabon; 6 Epidemiology, Biostatistics and Medical Informatic Service, Department of Fundamental and Mixed Sciences, Faculty of Medicine, University of Health Sciences, Libreville, Gabon; 7 Labor Für DNA-Analytik, Freiburg, Germany

**Keywords:** Biochemistry, Genetics, Molecular biology

## Abstract

Short tandem repeats (STRs) are repeating DNA sequences used in forensic human identity testing and the diagnosis of aneuploidies. Many STRs like Penta D and TPOX are used routinely for paternity tests, but these tests are not widely used in sub-Saharan Africa. In this study we recruited individuals from Gabonese families seeking a paternity test. After DNA extraction from buccal swabs, we genotyped samples using a panel of 22 STRs. A total of 115 unrelated subjects from 39 families were included. Allele frequencies of the 22 STR loci were determined in unrelated Gabonese subjects. The most polymorphic loci were D21S11 (16 alleles) and FGA (17 alleles), while D3S1358 and TH01 loci were less polymorphic, with five alleles each. Deviation from Hardy–Weinberg equilibrium was observed for TPOX, D3S1358, CSFPO and D7S820 loci. We reported tri-allelic patterns that indicate aneuploidies at a combined frequency of 4% (4/115) with 3% for Penta D (1/35) and 3% for TPOX (3/102). Furthermore, we identified a new tri-allelic genotype 5-8-16 for the Penta D locus located on chromosome 21 in a healthy subject. In addition, we observed three tri-allelic variants of TPOX, located on chromosome 2, in healthy subjects, namely 8-10-11, 8-9-10, and 8-8-10. Our study revealed unsuspected polymorphic variations in Penta D and TPOX for the first time in Gabon, raising several questions about chromosomal disorders. Further population genetics studies are needed in Gabon to better characterize these variations, both qualitatively and quantitative.

## Introduction

Advances in molecular biology have made it possible to identify genetic identification markers for common use. Among these markers, short tandem repeats (STRs) are used worldwide in paternity testing and to diagnose aneuploidies. STRs are repeating DNA sequences of 1–6 bp, where a difference in the number of repeating sequences results in different alleles and contributes to genetic polymorphisms at STR loci. Several loci, from 15 to 24 STRs, have been grouped into standard panels and are used routinely for paternity testing^1^.

Penta D is a simple pentanucleotide repeat (AAAGA) located on chromosome 21 (21q22.3), for which alleles ranging from 1.1 to 19 repeats have been observed by length-based genotyping, and alleles 5 to 19 have been sequenced^2^. Fifty-six Penta D STR alleles are reported in STRbase with 18 tri-allelic patterns^3^.

TPOX is a simple tetranucleotide repeat (AATG) located in the tenth intron of the human thyroid peroxidase gene on chromosome 2 (2p25.3). TPOX alleles size ranged from 4 to 18 repeats, and 22 tri-allelic patterns are reported in STRbase^4^. The tri-allelic pattern 8-10-11 is the most common^4^.

In 2004, Clayton et al*.* distinguished two types of tri-allelic patterns: tri-allelic Type 1 occurs when two peaks have a different height from the third and tri-allelic Type 2 occurs when all three peaks have a similar height^5^. More recently, Picanço et al*.* (2015) identified three tri-allelic Type 2 subcategories: Type 2-A (three peaks of the same height), Type 2-B (one peak with a 2:3 height ratio and one with 1:3 height ratio) and Type 2-C (one peak with a 3:3 height ratio)^6^.

In this study, we determined the allele frequencies of 22 STRs loci in unrelated Gabonese subjects, and we reported for the first time in the Gabonese population, the existence of a novel tri-allelic pattern of the Penta D locus, and tri-allelic patterns at the TPOX locus.

## Results

### Allele frequencies

We screened for paternity testing 115 unrelated subjects from 39 families at DNA-LAB Gabon located in Libreville. Were included 42 children, 39 presumed fathers and 34 mothers.

Of the 22 STRs tested, eleven were highly polymorphic (Table 1), while other loci had fewer than ten alleles (Table 1). The most polymorphic loci were D21S11 (16 alleles) and FGA (17 alleles), while D3S1358 and TH01 loci were less polymorphic, with only five alleles each. Deviations from Hardy–Weinberg equilibrium were observed in TPOX, D3S1358, CSFPO and D7S820 loci.

**Table 1:** Allele frequencies of the eleven (a) highly polymorphic, (b) lowly polymorphic STRs loci in unrelated Gabonese subjects.

(a) Highly polymorphic											
Alleles	D1S1656	D2S1338	FGA	F13A01	vWA	D12S391	PentaE	D18S51	D19S433	PentaD	D21S11
N	43	65	68	43	74	45	47	70	75	23	76
2.2										0.106	
3				0.012							
3.2				0.151							
4				0.047							
5				0.326			0.085			0.106	
6				0.116							
6.4							0.011				
7				0.221			0.191			0.021	
8				0.035			0.202	0.007		0.064	
9							0.011	0.007	0.007	0.170	
10	0.012			0.012			0.053			0.170	
11	0.058				0.007		0.053		0.120	0.170	
12	0.093			0.012			0.085	0.050	0.107	0.128	
12.2									0.027		
13	0.151			0.012	0.014		0.138	0.064	0.327	0.043	
13.2									0.033		
14	0.209	0.008		0.012	0.041		0.053	0.036	0.147		
14.2									0.067		
14.3	0.023										
15	0.128	0.015	0.007	0.023	0.216	0.167	0.053	0.179	0.053		
15.2									0.060		
15.3	0.012										
16	0.186	0.054		0.012	0.223	0.122	0.032	0.214	0.013	0.021	
16.2									0.033		
16.3	0.081										
17	0.012	0.092	0.007	0.012	0.236	0.156	0.021	0.121	0.007		
17.3	0.023					0.011					
18		0.038			0.122	0.256		0.150			0.007
18.2			0.007								
18.3	0.012					0.011					
19		0.138	0.036		0.088	0.044		0.086			
19.1					0.007						
20		0.077	0.044		0.041	0.122	0.011	0.036			
21		0.131	0.146		0.007	0.056		0.029			
22		0.208	0.190			0.033		0.014			
22.2			0.007								
23		0.077	0.204			0.022					
24		0.085	0.109					0.007			
24.2			0.007								
25		0.069	0.131								
26		0.008	0.058								
27			0.022								0.059
28			0.029								0.217
28.2											0.007
29			0.007								0.171
30											0.224
30.2											0.013
31											0.059
31.2			0.007								0.039
32											0.033
32.2											0.086
33											0.007
33.1											0.013
33.2											0.026
35											0.026
36											0.013
Ho	0.917	1	1	1	1	0.923	1	1	1	1	0.800
He	0.924	0.932	0.910	0.915	0.927	0.932	0.930	0.930	0.917	0.926	0.875
PD	0.989	0.991	0.985	0.987	0.990	0.991	0.991	0.991	0.988	0.990	0.973
PE	0.848	0.864	0.821	0.831	0.855	0.864	0.860	0.860	0.836	0.852	0.755
P	0.509	0.433	0.401	0.810	0.083	0.104	0.297	0.074	0.654	0.497	0.596

(b) Lowly polymorphic											
Alleles	TPOX	D3S1358	D5S818	CSFPO	SE33	D7S820	D8S1179	TH01	D13S317	D16S539	D22S1045
N	68	13	74	33	9	76	76	70	74	74	71
5										0.014	
6	0.066							0.107			
7	0.015			0.045		0.007		0.414			
8	0.358		0.041	0.076		0.158		0.264	0.007	0.014	
9	0.197		0.047	0.015		0.138	0.007	0.179	0.014	0.223	
9.3								0.036			
10	0.058		0.088	0.288		0.395	0.007		0.014	0.101	0.042
11	0.285		0.257	0.152		0.197	0.026		0.351	0.304	0.254
12	0.022		0.338	0.394		0.072	0.053		0.439	0.149	0.042
13			0.203	0.030		0.033	0.184		0.135	0.176	0.014
14		0.192	0.014				0.428		0.041	0.020	0.085
15		0.269	0.014		0.111		0.217				0.190
16		0.385			0.111		0.059				0.204
17		0.115			0.167		0.020				0.155
18		0.038			0.056						0.014
20					0.222						
21					0.056						
22					0.056						
25.2					0.111						
28.2					0.111						
Ho	0.769	0.909	1	0.909	1	0.750	0.909	0.917	0.900	0.900	0.875
He	0.902	0.934	0.930	0.938	0.921	0.924	0.926	0.917	0.905	0.885	0.898
PD	0.983	0.992	0.991	0.993	0.988	0.989	0.990	0.987	0.984	0.976	0.981
PE	0.807	0.868	0.860	0.876	0.844	0.848	0.852	0.835	0.812	0.773	0.799
P	0.000	0.024	0.449	0.018	0.137	0.001	0.093	0.321	0.565	0.729	0.373

Ho (observed heterozygosity), He (expected heterozygosity), PD (power of discrimination), PE (probability of exclusion), P (Hardy–Weinberg equilibrium exact test).

Comparing 10 STRs, the allelic frequencies observed in our study were not statistically different from those of the Gabonese samples in a previous study published in 2002 (Table 2)^11^. Furthermore, comparison of the frequencies of 13 STRs between African-Americans from California (USA)^15^ and the Gabonese in the present study also revealed no significant differences (Table 3).

**Table 2:** 20-year comparisons between allele frequencies of STR loci in Gabonese subjects using Wilcoxon signed rank test with continuity correction.

Tested STR	Allele	Frequency among Gabonese subjects in our study (year 2023)	Frequency among Gabonese subjects in the study published in 2002^11^	P
D2S1338	14	0.008	0.000	0.84
	15	0.015	0.019	
	16	0.054	0.069	
	17	0.092	0.093	
	18	0.038	0.102	
	19	0.138	0.176	
	20	0.077	0.130	
	21	0.131	0.083	
	22	0.208	0.097	
	23	0.077	0.060	
	24	0.085	0.065	
	25	0.069	0.079	
	26	0.008	0.028	
D3S1358	12	0.000	0.005	0.80
	13	0.000	0.005	
	14	0.192	0.088	
	15	0.269	0.342	
	16	0.385	0.310	
	17	0.115	0.208	
	18	0.038	0.042	
FGA	15	0.007	0.000	0.75
	17	0.007	0.014	
	18	0.000	0.014	
	18.2	0.007	0.014	
	19	0.036	0.055	
	19.2	0.000	0.005	
	20	0.044	0.060	
	21	0.146	0.106	
	21.2	0.000	0.005	
	22	0.190	0.129	
	22.2	0.007	0.000	
	23	0.204	0.171	
	24	0.109	0.190	
	24.2	0.007	0.000	
	25	0.131	0.157	
	26	0.058	0.037	
	27	0.022	0.028	
	28	0.029	0.005	
	29	0.007	0.000	
	31.2	0.007	0.005	
	44	0.000	0.005	
D8S1179	9	0.007	0.000	1
	10	0.007	0.000	
	11	0.026	0.056	
	12	0.053	0.139	
	13	0.184	0.143	
	14	0.428	0.347	
	15	0.217	0.241	
	16	0.059	0.074	
	17	0.020	0.000	
TH01	6	0.107	0.093	1
	7	0.414	0.389	
	8	0.264	0.319	
	9	0.179	0.134	
	9.3	0.036	0.051	
	10	0.000	0.014	
vWA	11	0.007	0.014	0.79
	13	0.014	0.023	
	14	0.041	0.079	
	15	0.216	0.217	
	16	0.223	0.255	
	17	0.236	0.157	
	18	0.122	0.176	
	19	0.088	0.060	
	19.1	0.007	0.000	
	20	0.041	0.014	
	21	0.007	0.005	
D16S539	5	0.014	0.009	1
	8	0.014	0.032	
	9	0.223	0.278	
	10	0.101	0.144	
	11	0.304	0.255	
	12	0.149	0.134	
	13	0.176	0.139	
	14	0.020	0.009	
D18S51	8	0.007	0.000	0.45
	9	0.007	0.000	
	11	0.000	0.005	
	12	0.050	0.014	
	12.2	0.000	0.005	
	13	0.064	0.018	
	13.2	0.000	0.005	
	14	0.036	0.042	
	15	0.179	0.157	
	16	0.214	0.194	
	17	0.121	0.227	
	18	0.150	0.153	
	19	0.086	0.092	
	20	0.036	0.051	
	21	0.029	0.023	
	22	0.014	0.009	
	24	0.007	0.005	
D19S433	9	0.007	0.009	0.93
	10	0.000	0.037	
	11	0.120	0.116	
	12	0.107	0.088	
	12.2	0.027	0.055	
	13	0.327	0.241	
	13.2	0.033	0.088	
	14	0.147	0.166	
	14.2	0.067	0.065	
	15	0.053	0.028	
	15.2	0.060	0.069	
	16	0.013	0.005	
	16.2	0.033	0.028	
	17	0.007	0.000	
	17.2	0.000	0.005	
D21S11	18	0.007	0.000	1
	27	0.059	0.093	
	28	0.217	0.264	
	28.2	0.007	0.000	
	29	0.171	0.162	
	30	0.224	0.171	
	30.2	0.013	0.009	
	31	0.059	0.097	
	31.2	0.039	0.042	
	32	0.033	0.018	
	32.1	0.000	0.009	
	32.2	0.086	0.032	
	33	0.007	0.009	
	33.1	0.013	0.014	
	33.2	0.026	0.014	
	35	0.026	0.042	
	35.1	0.000	0.005	
	35.2	0.000	0.005	
	36	0.013	0.000

**Table 3:** Comparisons between allele frequencies of STR loci in Gabonese subjects of our study and African-American subjects from California using Wilcoxon signed rank test with continuity correction.

Tested STR	Allele	Frequency among Gabonese subjects in our study (year 2023)	Frequency among African-Americans from California^15^	P
TPOX	6	0.066	0.0625	1
	7	0.015	0.0250	
	8	0.358	0.3325	
	9	0.197	0.2100	
	10	0.058	0.0600	
	11	0.285	0.2700	
	12	0.022	0.0400	
D3S1358	< 12	0.000	0.0025	0.64
	12	0.000	0.0050	
	13	0.000	0.0125	
	14	0.192	0.0850	
	15	0.269	0.2750	
	16	0.385	0.3625	
	17	0.115	0.2050	
	18	0.038	0.0500	
	19	0.000	0.0025	
FGA	15	0.007	0.0025	0.78
	17	0.007	0.0050	
	18.2	0.007	0.0150	
	19	0.036	0.0675	
	19.2	0.000	0.0075	
	20	0.044	0.0450	
	20.2	0.000	0.0025	
	21	0.146	0.0950	
	22	0.190	0.1750	
	22.2	0.007	0.0025	
	23	0.204	0.1975	
	24	0.109	0.1625	
	24.2	0.007	0.0000	
	25	0.131	0.1125	
	26	0.058	0.0425	
	27	0.022	0.0350	
	28	0.029	0.0175	
	29	0.007	0.0000	
	31.2	0.007	0.0150	
D5S818	7	0.000	0.0050	1
	8	0.041	0.0525	
	9	0.047	0.0175	
	10	0.088	0.0600	
	11	0.257	0.2825	
	12	0.338	0.3575	
	13	0.203	0.2075	
	14	0.014	0.0100	
	15	0.014	0.0050	
	> 15	0.000	0.0025	
CSFPO	7	0.045	0.0550	0.23
	8	0.076	0.0700	
	9	0.015	0.0350	
	10	0.288	0.3125	
	11	0.152	0.2250	
	12	0.394	0.2425	
	13	0.030	0.0500	
	14	0.000	0.0100	
D7S820	7	0.007	0.0050	0.95
	8	0.158	0.2325	
	9	0.138	0.1125	
	10	0.395	0.3400	
	11	0.197	0.2175	
	12	0.072	0.0775	
	13	0.033	0.0100	
	14	0.000	0.0050	
D8S1179	< 9	0.000	0.0050	0.65
	9	0.007	0.0025	
	10	0.007	0.0075	
	11	0.026	0.0625	
	12	0.053	0.1075	
	13	0.184	0.2250	
	14	0.428	0.3100	
	15	0.217	0.1975	
	16	0.059	0.0575	
	17	0.020	0.0250	
TH01	5	0.000	0.0050	0.69
	6	0.107	0.1175	
	7	0.414	0.4250	
	8	0.264	0.1875	
	9	0.179	0.1350	
	9.3	0.036	0.1225	
	10	0.000	0.0075	
vWA	11	0.007	0.0000	0.96
	13	0.014	0.0125	
	14	0.041	0.0625	
	15	0.216	0.1925	
	16	0.223	0.2525	
	17	0.236	0.2450	
	18	0.122	0.1325	
	19	0.088	0.0725	
	19.1	0.007	0.0000	
	20	0.041	0.0150	
	21	0.007	0.0100	
	> 21	0.000	0.0050	
D13S317	8	0.007	0.0450	0.81
	9	0.014	0.0325	
	10	0.014	0.0225	
	11	0.351	0.2700	
	12	0.439	0.4050	
	13	0.135	0.1525	
	14	0.041	0.0725	
D16S539	5	0.014	0.0025	0.69
	8	0.014	0.0400	
	9	0.223	0.1825	
	10	0.101	0.1050	
	11	0.304	0.3100	
	12	0.149	0.2075	
	13	0.176	0.1300	
	14	0.020	0.0225	
D18S51	8	0.007	0.0000	0.36
	9	0.007	0.0050	
	11	0.000	0.0075	
	12	0.050	0.0625	
	13	0.064	0.0475	
	13.2	0.000	0.0050	
	14	0.036	0.0625	
	14.2	0.000	0.0100	
	15	0.179	0.1875	
	16	0.214	0.1725	
	17	0.121	0.1500	
	18	0.150	0.0950	
	19	0.086	0.0975	
	20	0.036	0.0425	
	21	0.029	0.0350	
	22	0.014	0.0175	
	24	0.007	0.0025	
D21S11	18	0.007	0.0000	0.69
	26	0.000	0.0025	
	26.2	0.000	0.0025	
	27	0.059	0.0375	
	28	0.217	0.2850	
	28.2	0.007	0.0000	
	29	0.171	0.2000	
	29.2	0.000	0.0025	
	30	0.224	0.1500	
	30.2	0.013	0.0225	
	31	0.059	0.0700	
	31.2	0.039	0.0375	
	32	0.033	0.0100	
	32.2	0.086	0.0800	
	33	0.007	0.0025	
	33.1	0.013	0.0050	
	33.2	0.026	0.0350	
	34	0.000	0.0100	
	34.1	0.000	0.0025	
	34.2	0.000	0.0025	
	35	0.026	0.0350	
	36	0.013	0.0075	

### Tri-allelic patterns

We found tri-allelic patterns in 8% of recruited families (**3**/39 families), and 4% of recruited subjects (**4**/115 subjects). In all tri-allelic cases, presumed fathers were the biological fathers of tested children, and we did not observe any physical abnormalities that could suggest a genetic disorder in any member of the recruited families.

### Penta D family case

**Figure 1:**
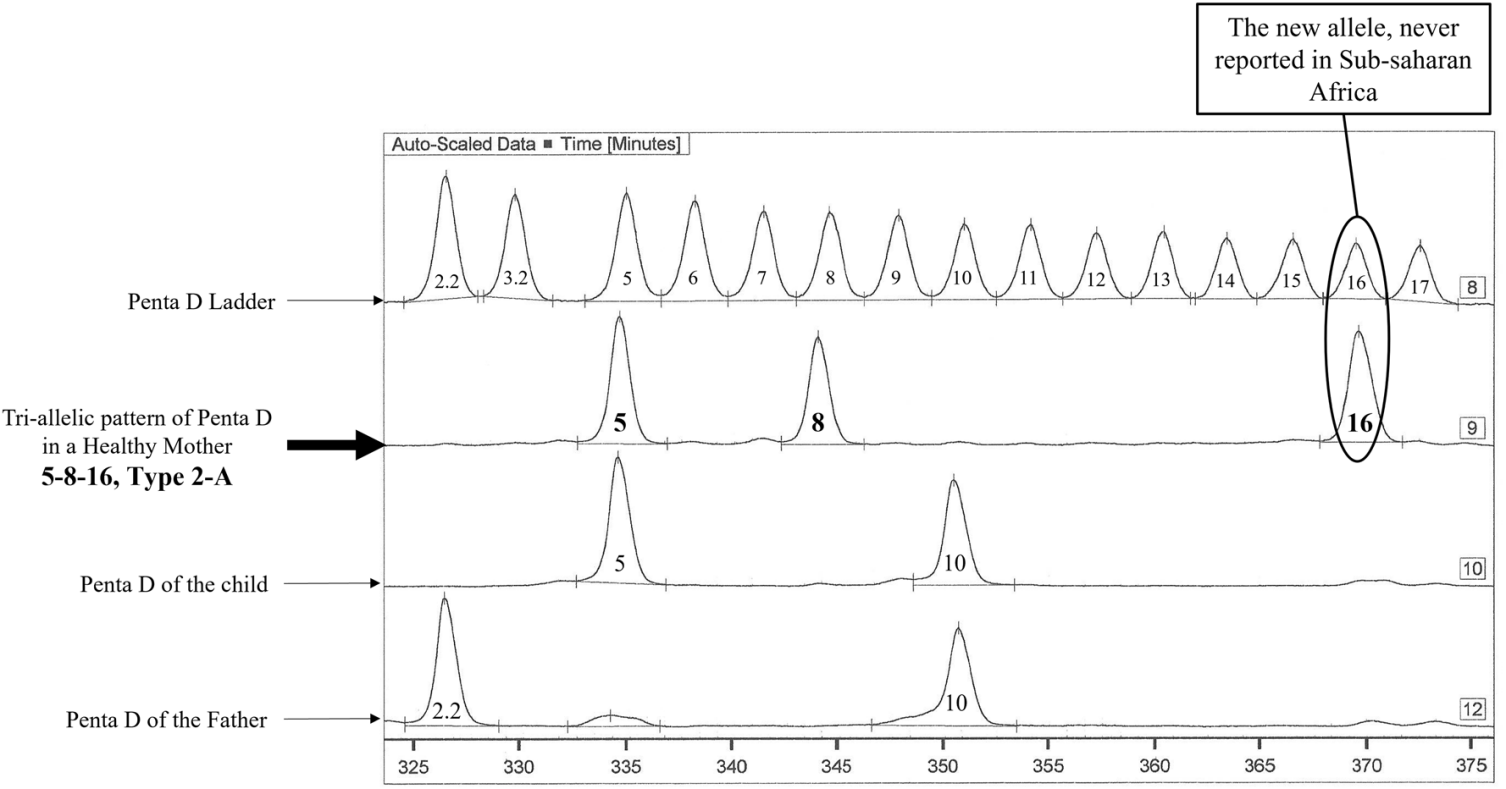
Penta D tri-allelic genotype observed in a Gabonese family (depicted by Pharmacia DNA Fragment Manager V1.2 software).

Were screened for parentage a phenotypically normal family. Penta D genotypes were 2.2–10 for presumed father, 5–8-16 for the mother and 5–10 for the child. This new tri-allelic Penta D genotype 5-8-16 was observed with a frequency of 3% in tested subjects (1/35 subjects) (Fig. 1). This genotype has never been reported in Sub-Saharan Africa.

### TPOX family cases

**Figure 2:**
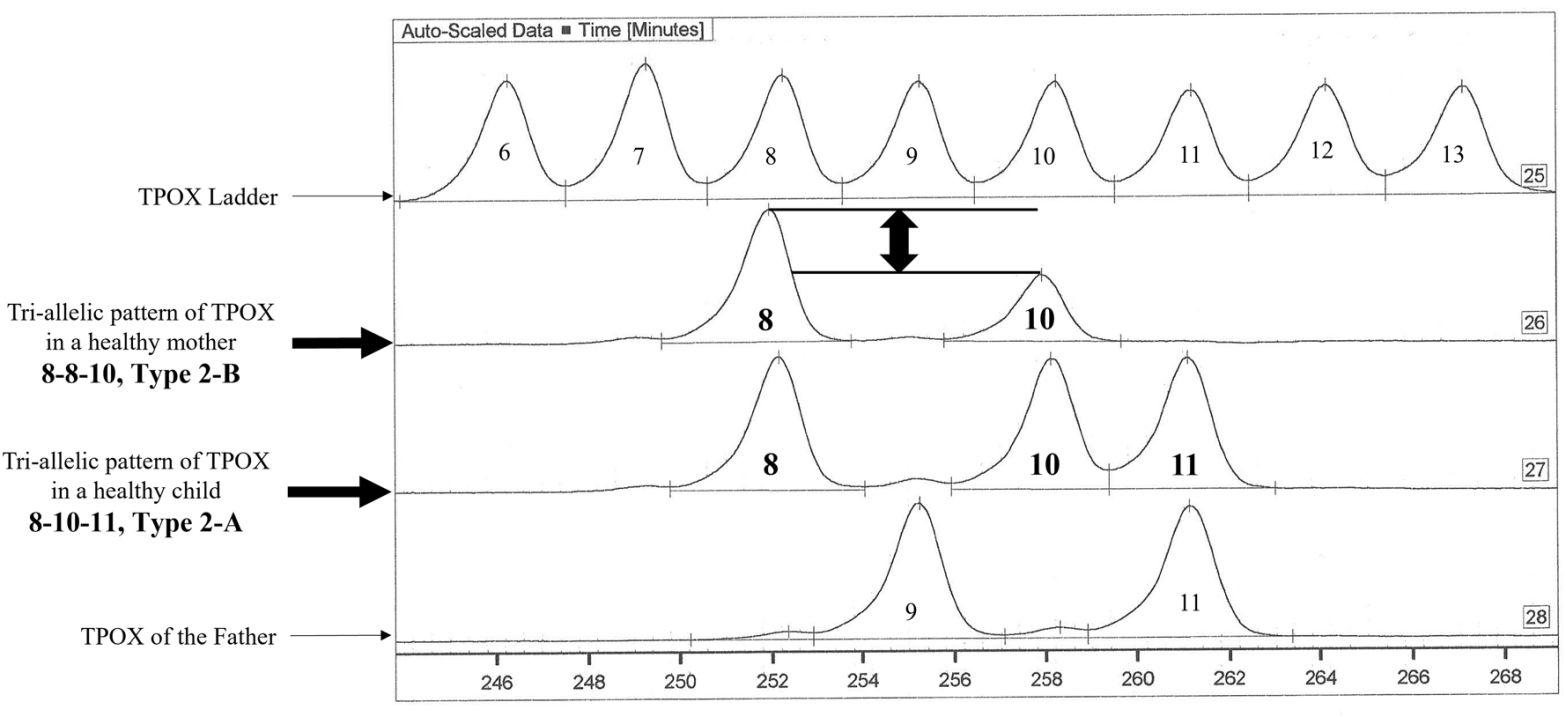
TPOX tri-allelic patterns observed in healthy Gabonese family (depicted by Pharmacia DNA Fragment Manager V1.2 software).

Tri-allelic TPOX genotypes were observed with a frequency of 3% (3/102 subjects tested). In the second family, which comprised a man with genotype 9-11, a woman with genotype 8-8-10, and their child (male) with genotype 8-10-11, we found two different types of tri-allelic TPOX genotypes. In this family, the tri-allelic type 2-B, 8-8-10, was observed in the healthy mother (Fig. 2). Type 2-A was found in the healthy child of this family (8-10-11, Fig. 2) as in the healthy child of the third family (8–9-10; figure not show). The third family comprising a father with genotype 8-8, a mother with genotype 9-9, and their child (female) with genotype 8-9-10 (figure not show).

## Discussion

We investigated more loci than in a previous study of the Gabonese population twenty years ago^11^. Allele frequencies were similar between the two studies, but we found features related to specific alleles that did not appear in the previous survey. These were allele 14 of D2S1338, alleles 15, 22.2, 24.2, and 29 of FGA, alleles 9, 10, and 17 of D8S1179, allele 19.1 of vWA, alleles 8 and 9 of D18S51, allele 17 of D19S433, and alleles 18, 28.2 and 36 of D21S11. All are at low frequency in our study. In contrast, alleles 12 and 13 of D3S1358, alleles 18, 19.2, and 21.2 of FGA, allele 10 of TH01, alleles 11, 12.2, and 13.2 of D18S51, alleles 10 and 17.2 of D19S433, and alleles 32.1, 34, 35.1 and 35.2 of D21S11 were absent in our study but appeared in the earlier survey^11^. The polymorphic loci FGA, D18S51, and D21S11 showed remarkable differences in terms of various allele presence. Locus D8S1179 was the most polymorphic in our study, with three new alleles compared to the older study, of which one of the alleles (10) is involved in tri-allelic inheritance in India^12^.

Power discrimination analysis suggests that all 22 STR may be promising markers for paternity testing (PD between 0.973 and 0.993). One of the four probability tests showing significant departures from the HWE equilibrium (CSFPO locus) was also significant in Rwandans (Hutu) and Angolans^13,14^. The frequency of homozygotes at the CSFPO locus (9/33 = 27%) indicates that this deviation is due to homozygote excess, as in Rwandans^13^. The allele frequencies are also similar to those for descendants of African people in California^15^. These comparisons suggest that these loci are stable and good genetic identification indicators.

We report a tri-allelic Penta D pattern (5–8-16) that has not been described previously. The Penta D allele 5, the only allele transmitted from the mother to the child in family #1, occurs at a frequency of 11% in our study and 4% in Africa^16^. Of the other Penta D alleles in this family, allele 8, is less frequent in our study population (6%) than in Africa more generally (15.5%)^16^. Penta D allele 16, at a frequency of 2% in our study, has not yet been detected elsewhere in Africa but exists in other populations, such as the Middle East, at a frequency of 2.5%^16^. Further population studies of the STR locus Penta D should be conducted in Sub-Saharan Africa to determine the types of changes and their frequencies. In our study, the phenotypically normal mother has a tri-allelic pattern of Penta D. The other STR on chromosome 21 in the panels of STR loci used for paternity testing, D21S11, showed no allelic abnormality. Her tested offspring inherited only two alleles, one from each parent. A tri-allelic pattern of Penta D indicates a genetic abnormality on chromosome 21. Trisomy 21 (also known as Down syndrome) is the most common chromosomal anomaly and corresponds to the presence of an extra chromosome 21, in whole or in part. It can be due to various chromosomal aberrations: free trisomy, translocations, mosaicism, critical region duplication, and other structural rearrangements of chromosome 21^17^. Mosaicism or partial trisomy 21 are more challenging to diagnose because the karyotype is often normal^17^, showing the importance of studies of Penta D STR.

The tri-allelic TPOX genotypes we observed were tri-allelic Type 2, which is due to a constitutional chromosomal rearrangement, while tri-allelic Type 1 is probably due to a mutation in an early somatic cell^5^. The tri-allelic Type 2 pattern of TPOX is present at a very low frequency in various human populations, ranging from 0.003 to 0.2%^18^. The highest frequency of tri-allelic TPOX genotypes observed (2.4% or 165/6827 people) was in indigenous black populations from South Africa^19^. Although our sample size was small, the observed frequency (3% or 3/102 people investigated) supports observations in African populations^19^. Moreover, the presence of tri-allelic genotypes of TPOX in Gabon (Central Africa) supports the hypothesis that the TPOX variants may have existed before the expansion of Bantus from Central Africa^19,20^.

In the second family, a mother with three TPOX alleles transmitted two of them to her son, supporting the hypothesis that the extra allele comes from the X chromosome, as proposed by two studies^6,19^, or from chromosome 2 with a potential impact of chromosomal rearrangement on the activity of Y-sperm^21^.

In the third family, the pattern is entirely different. Each parent transmitted an allele to their daughter (allele 8 from the mother and allele 9 from the father), but the daughter shows an extra allele de novo, allele 10. Some authors have suggested that the additional allele in TPOX is allele 10^6,19^. However, other authors have shown that it is allele 11 in Chinese and Korean populations^21^. Allele 11 results from a strand slippage mutation of an extra allele 10 of TPOX originating from Bantu groups in Africa^21^. Our results show that allele 10 is an additional allele, as observed in the TPOX locus of the daughter in the third family. Furthermore, this allele was found in other tri-allelic TPOX genotypes in our study: a healthy mother and her healthy child (from the second family).

## Limitations of the study and future directions

Due to a lack of funding, we could not sequence the extra-allele 10 of TPOX found in Family #3. This was the main limitation of this work. Future studies could sequence the de novo allele in this girl or extend population genetic study to her entire family.

## Conclusion

We observed similar allele frequencies of 22 STRs to those in other Black populations. These findings suggest that these STRs are good identification markers, allowing us to diagnose aneuploidies without symptoms. The presence in chromosome 21 of a tri-allelic genotype of the Penta D locus with a new allele in our study suggests that we need more in-depth studies of this locus in sub-Saharan Africa. The presence of three subtypes (8-8-10, 8-10-11, and 8-9-10) of the tri-allelic variants of TPOX in our small sample suggests that we need an extended study of genetic polymorphism in Central Africa, where the Bantu peoples originate.

## Methods

Data were collected during paternity tests on indigenous Gabonese people. As such analyses are not yet routine in Gabon, we collaborated with a partner laboratory (Labor Für DNA Analytik/Germany) for the complete analysis after DNA extraction. On this topic, DNA was extracted from buccal swabs and prepared with the nucleospin tissue kit following the manufacturer's protocol (Macherey Nagel, Freiburg, Germany). This form clearly states that the signatory parties agreed to use the results for research and publications.

To assess allelic frequencies in this study, we only considered unrelated subjects who were therefore defined in two ways. For 2-parent families, all children were excluded from the unrelated subject’s group. In the case of single-parent families, we included in this group, children without proven parentage with the presumed father.

For the purposes of this study, twenty-two STRs were used (D1S1656, TPOX, D2S1338, D3S1358, FGA, D5S818, CSFPO, F13A01, SE33, D7S820, D8S1179, TH01, vWA, D12S391, D13S317, Penta E, D16S539, D18S51, D19S433, Penta D, D21S11, D22S1045). Primers were ordered from TibMolBiol, Berlin (Germany) according to reference sequences for the 22 STR loci available at Ref.^7^. We labelled one primer of each primer pair in yellow (5'fluorescein). Alleles were determined based on allelic ladders. To get allelic ladders, we have amplified the Powerplex 2.1 ladder from Promega (Walldorf, Germany). Each ladder was checked with commercially available K562 and M2800 DNA (Promega, Walldorf, Germany). Finally, 0.3–0.5 µl of the PCR reaction were loaded on an acrylamide gel. For gel preparation, the Rotiphorese Sequenzier-Gel system from Carl-Roth GmbH, Karlsruhe (Germany) was used (Cat. No. A431). Based on Labor für DNA-Analytik results, likelihood ratio values were calculated according to Ref.^8^.

### Ethics approval and consent to participate and for publication

This study was conducted according to the Declaration of Helsinki^9^ and the legislation in force in the Gabonese Republic. The Scientific Council of the Mother and Child University Hospital—Jeanne Ebori Foundation of Libreville, in charge of ethics approval in this medical facility, approved the study. Furthermore, Consent was obtained from all participants. Before any sampling in the context of parentage screening, the fathers and mothers give us their written consent by signing the consent form of DNA-LAB-Gabon dedicated to this purpose, preceded by the words "read and approved". This form clearly states in point 4 that the different signatory parties agreed that the results are used for research and publications. Finally, if the child was a minor, all parentage tests were carried out with the written consent of the father, mother and/or legal guardian. Where applicable, tests were conducted by decision of the court of first instance of the city of residence.

### Statistical analysis

Data were compiled using Microsoft Excel 2013, and the database was analysed using R version 4.2.2. We used the Wilcoxon signed rank test with continuity correction to compare allele frequencies of STR loci between Gabonese subjects from 2002 and 2023; and between Gabonese subjects and African-American subjects. Values of p < 0.05 were considered statistically significant. Allele frequencies were calculated from the numbers of each genotype obtained in the sample set of unrelated subjects. Expected heterozygosity (He), power of discrimination (PD), probability of exclusion (PE), and exact tests of Hardy–Weinberg equilibrium were all performed using EasyDNA software (https://saasweb.hku.hk/EasyDNA/)^10^.

## Data Availability

The datasets used and/or analysed during the current study are available from the corresponding author upon reasonable request.
